# Integrated Process for Bioenergy Production and Water Recycling in the Dairy Industry: Selection of *Kluyveromyces* Strains for Direct Conversion of Concentrated Lactose-Rich Streams into Bioethanol

**DOI:** 10.3390/microorganisms7110545

**Published:** 2019-11-09

**Authors:** Maria José Leandro, Susana Marques, Belina Ribeiro, Helena Santos, César Fonseca

**Affiliations:** 1 Unidade de Bioenergia, Laboratório Nacional de Energia e Geologia, I.P. (LNEG), Estrada do Paço do Lumiar 22, 1649-038 Lisboa, Portugal; mjose.leandro@itqb.unl.pt (M.J.L.); susana.marques@lneg.pt (S.M.); belina.ribeiro@lneg.pt (B.R.); 2 Instituto de Tecnologia Química e Biológica António Xavier, Biology Division, Universidade Nova de Lisboa, Av. da República, 2780-157 Oeiras, Portugal; santos@itqb.unl.pt; 3 Department of Chemistry and Bioscience, Section for Sustainable Biotechnology, Aalborg University, A. C. Meyers Vænge 15, 2450 Copenhagen, Denmark

**Keywords:** dairy industry, *Kluyveromyces*, lactose fermentation, bioethanol, water recycling

## Abstract

Dairy industries have a high environmental impact, with very high energy and water consumption and polluting effluents. To increase the sustainability of these industries it is urgent to implement technologies for wastewater treatment allowing water recycling and energy savings. In this study, dairy wastewater was processed by ultrafiltration and nanofiltration or ultrafiltration and reverse osmosis (UF/RO) and retentates from the second membrane separation processes were assessed for bioenergy production. Lactose-fermenting yeasts were tested in direct conversion of the retentates (lactose-rich streams) into bioethanol. Two *Kluyveromyces* strains efficiently fermented all the lactose, with ethanol yields higher than 90% (>0.47 g/g yield). Under severe oxygen-limiting conditions, the *K. marxianus* PYCC 3286 strain reached 70 g/L of ethanol, which is compatible with energy-efficient distillation processes. In turn, the RO permeate is suitable for recycling into the cleaning process. The proposed integrated process, using UF/RO membrane technology, could allow water recycling (RO permeate) and bioenergy production (from RO retentate) for a more sustainable dairy industry.

## 1. Introduction

According to the EU, agricultural markets outlook report for 2017–2030, the world milk production will surpass one billion tons/year by 2030. Moreover, strong growth of the dairy processing industry is foreseen, as consumption of cheese and processed dairy products is expected to rise [1]. Consequently, huge amounts of dairy effluent streams need to be handled, typically carrying out polluting charges of 0.4 to 8.0 g/L BOD_5_ [2].

The dairy industry can use as much as 60 L of water per kg of processed milk, most of the water being consumed in the clean-in-place stages [3]. Dairy wastewaters mainly contain salts, lactose, proteins, and lipids, and water recycling is mandatory to increase dairies sustainability. 

The most promising treatment technologies for water recycling are based on membrane separation processes. Nanofiltration (NF) and reverse osmosis (RO) are pressure driven membrane separation processes currently employed to remove organic components from wastewaters, producing, as permeate, purified water to be recycled in the cleaning process [3,4]. The nanofiltration membranes contain pores that act like a sieve, allowing the selective passage of components depending on their shape and molecular size. On the one hand, because of their efficiency for the removal of organic compounds, relatively low-pressure requirements, and selective separation of salts, nanofiltration membranes are increasingly employed in wastewater treatment [5]. On the other hand, membranes used in reverse osmosis are not porous and can be considered as “water-swollen gels”, allowing the diffusion of water and vestiges of inorganic ions towards the low-pressure side, while retaining most molecules and ions (e.g., chloride). Indeed, reverse osmosis is one of the most economically advantageous processes used for water purification and recovery of valuable compounds in several types of industrial waste streams [6,7].

The major components of dairy effluents are protein and lactose. Proteins from whey are often recovered through ultrafiltration (UF) processes and sold as food supplements (whey protein powder) [8] or microparticulated and incorporated into dairy products to improve texture. Membrane separation technologies might be further applied for treatment of dairy wastewaters to reduce polluting discharge or to recycle water according to local law regulations. It has been reported that the water recovered from whey, by using a combined ultrafiltration and reverse osmosis system, exhibits a cleaning efficiency similar to that of fresh potable water, without affecting the quality or safety of the manufactured product [9]. The use of UF prior to NF or RO, not only allows protein recovery but also reduces membrane fouling during the NF and RO processes. In addition, the possibility of using the NF or RO retentates as a substrate for the production of bioenergy carriers, such as biogas and bioethanol, will boost the economic competitiveness of the industrial process.

*Saccharomyces cerevisiae* is widely used for the industrial production of bioethanol. Wild-type strains are not able to ferment lactose because they lack lactose transporters or beta-galactosidase, however, they are able to ferment d-glucose and d-galactose, the sugars resulting from lactose hydrolysis. On the one hand, the inclusion of a previous acid hydrolysis step can lead to the formation of fermentation inhibitors; on the other hand, an enzymatic hydrolysis step will increase process costs. The use of recombinant *S. cerevisiae* strains, engineered for lactose fermentation by expressing heterologous lactose permease or beta-galactosidase genes, is a possible alternative [10,11], but the use of genetically modified organisms in the food sector raises particular concerns.

Another option is to use natural lactose-fermenting yeasts belonging to the genus *Kluyveromyces*, namely *K. lactis* and *K. marxianus*, already used in some fermented dairy products. Unlike *S. cerevisiae*, these are Crabtree-negative yeasts, meaning that under full aerobic conditions respiration is predominant [12]. Conversely, under oxygen-limiting conditions fermentation is preferred with ethanol as the main product. *K. marxianus* can grow over a broader range of temperatures than *K. lactis*, tolerating temperatures as high as 45 °C [13]. Their GRAS (generally regarded as safe) status, together with thermotolerance and high growth and fermentation yields, constitute desirable traits for industrial applications. These yeasts have been used to convert lactose from cheese whey into yeast biomass (animal feed additive), bio-ingredients (e.g., oligonucleotides, used as flavor enhancers), and enzymes, namely beta-galactosidase for lactose-free milk [14,15].

The European Project “SUSMILK Re-design of the dairy industry for sustainable milk processing” (FP7-613589) is aimed at developing a sustainable dairy industry. In the framework of this project, wastewaters were processed by UF, to obtain microparticulated whey protein concentrates (UF retentates), followed by NF or RO to generate permeates to be recycled into the cleaning process, and retentates with high lactose content (>100 g/L), here assessed for the production of bioethanol. The performance of ten *Kluyveromyces* strains was evaluated, at high-cell density, without supplementation, in order to identify efficient yeasts for industrial bioethanol production from concentrated lactose-rich streams. Two strains were selected exhibiting fermentation performances superior to those of the *K. marxianus* CBS 397 strain, thus far regarded as one of the most efficient lactose-fermenting yeasts for industrial lactose-fermenting processes.

## 2. Materials and Methods

### 2.1. Source and Characterization of Nanofiltration (NF) and Reverse Osmosis (RO) Permeates and Retentates

Cheese whey was obtained from Queizuar (Queixerías Bama, A Coruña, Spain), processed by cross flow ultrafiltration (Membralox^®^ EP 1940), at permeate rates of 5000 to 10,000 L/day, and the generated permeates further processed by cross flow nanofiltration (DOW^TM^ Filmtec NF 3840/30) or reverse osmosis (DOW^TM^ Filmtec RO 3838/30) by Universidad de Santiago de Compostela, Aula de Productos Lácteos, Spain. The filtration conditions for both NF and RO were the following: feed rate, 8000 L/h; permeate flow rate, 5800 L/h; and transmembrane pressure, 25 to 33 bar. NF and RO retentates were sterilized in autoclave (15 min at 121 °C), centrifuged (12,857× *g* at 4 °C for 20 min) to remove precipitated components and stored at 4 °C. The NF and RO permeates and processed retentates were analyzed for pH and ionic, carbohydrate, and protein characterization.

Physicochemical analysis of permeates and processed retentates were carried out by the Mineral Science and Technology Unit of LNEG (S. Mamede de Infesta, Porto, Portugal). The pH was determined by potentiometry. Cations, lithium, sodium, potassium, magnesium, calcium, iron, manganese, copper, and zinc were determined by flame atomic absorption spectroscopy; ammonium by potentiometry; cadmium and lead by plasma optical emission spectroscopy (ICP-OES); and mercury by inductively coupled plasma mass spectrometry (ICP-MS). Anions, fluoride, chloride, nitrate, phosphate, and sulfate were quantified by ionic chromatography; nitrite by visible molecular absorption spectrometry; and bicarbonate by volumetric titration. 

Carbohydrates were quantified in a high-performance liquid chromatography (HPLC) system Waters Alliance 2695 Separation Module (Waters Corporation, Milford, MA, USA), equipped with a Waters 2414 Refractive Index Detector and a Waters 486 Tunable Absorbance Detector (210 nm). An Aminex HPX-87H column (Bio-Rad Laboratories, Hercules, CA, USA) was used to separate the compounds at 60 °C with 5 mM sulfuric acid as mobile phase at a flow rate of 0.5 mL/min. Protein content of processed retentates was determined by Pierce™ BCA Protein Assay kit.

### 2.2. Yeast Strains and Fermentation Assays

The *Kluyveromyces* strains (Table 1) were obtained from the Portuguese Yeast Culture Collection (PYCC), Caparica, Portugal. Strains were routinely maintained in rich YPD medium (20 g/L peptone, 10 g/L yeast extract, and 20 g/L d-glucose).

The fermentation process was performed in shake flasks (volume ratio medium/flask 1/5), with cotton plugs (for mild oxygen-limiting conditions) or rubber plugs with piercing needles with 0.22 μm pore size cellulose acetate filter, for pressure release (for severe oxygen-limiting conditions), in an orbital shaker (Agitorb 200, Aralab) at 30 °C and 150× rpm. The *Kluyveromyces* strains were grown for approximately 24 h in YPD medium. Cells were harvested by centrifugation (10,414× *g* at 4 °C for 10 min), washed twice with cold sterile water and used to inoculate 20 mL sterile NF or RO retentates, at an initial biomass of 4 to 5 g cell dry weight per liter (gCDW/L). The fermentation assays were performed in triplicate.

The fermentation process was monitored by periodic sampling. Cell growth was monitored by optical density (OD) measurements at 600 nm in a DU^®^ 800 Spectrophotometer Beckman Coulter and by determination of cell dry weight (CDW), after 24 h drying at 100 °C. For the analysis of substrate consumption and product formation, samples were centrifuged (16,100× *g*) for 5 min at 4 °C and supernatants were stored at −20 °C. Thawed supernatants were diluted (1/20) with 5 mM sulfuric acid, incubated for 15 min at 60 °C and 15 min at room temperature. Precipitated proteins were removed by centrifugation (16,100× *g* for 10 min at 4 °C) and the resulting supernatants were filtered through a 0.22 μm pore size nylon filter and analyzed by HPLC as described in the previous section.

## 3. Results and Discussion

### 3.1. Chemical Composition of Nanofiltration and Reverse Osmosis Permeates and Retentates from Dairy Wastewater

The processing of industrial water streams through nanofiltration (NF) or reverse osmosis (RO) has been contemplated towards recycling of water and chemicals or disposal according to regulations. Wastewater from the dairy industry is usually a diluted version of milk, dairy products, or by-products (e.g., whey), having lactose concentrations equal or inferior to 50 g/L. Processing wastewater from the dairy industry through membrane technology contributes to the selective recovery of chemicals. UF can retain the majority of proteins, while NF or RO can concentrate the remaining organic chemicals, with lactose as the major component. This work is based on processing dairy wastewaters through UF, to recover the majority of the proteins in the retentate, followed by UF permeate processing through NF or RO. The permeates and retentates obtained from the NF or RO processing were analyzed, targeting specific applications, permeates for water recycling into the cleaning process and retentates for the production of bioethanol. With this purpose, processing of wastewater through UF/NF or UF/RO was designed to obtain a lactose concentration of 120 to 150 g/L in the retentates, in order to reach, by yeast fermentation, ethanol titers higher than 50 g/L, which is compatible with energy-efficient distillation processes [16].

#### 3.1.1. Composition of NF and RO Permeates and Comparison with Drinking Water Regulation

The permeates from the NF or RO processing were analyzed in relation to pH, conductivity, total dissolved solids, anions, cations, and vestigial elements (Table 2) against the following current recommendations and regulations for drinking water: Guidelines for drinking-water quality [17], EU Directive 98/83/EC on the quality of water intended for human consumption [18], Regulation on ground water and drinking water [19] and Water quality standards of drinking water [20] (Table S1 in Supplementary Materials). As expected, NF revealed lower salt rejection than RO, leading to higher total dissolved solids and conductivity in the permeate (3.86 vs. 0.19 g/L and 6440 vs. 317 μS/cm, respectively). The conductivity limit defined by the EU Directive 98/83/EC for drinking water is 2500 μS/cm and the total dissolved solids are generally recommended to be below 0.5 to 0.6 g/L, both limits only met by RO permeate. Both of the NF and RO permeates are acidic (pH of 5.85 and 5.39, respectively) exhibiting pH values outside the limits normally suggested (6.5 < pH < 8.5). Nevertheless, pH adjustments can easily be performed if necessary.

Among the most hazardous inorganic chemicals are the anions fluoride and nitrate/nitrite. Fluoride and nitrate/nitrite levels in both NF and RO permeate are within the limits defined by the different regulations for drinking-water quality. Chloride and sulfate have recommended limits defined only due to aesthetic (odor and taste) effects (<200–250 mg/L and <250–500 mg/L, respectively). While chloride in RO permeate (78.5 mg/L) is within the limits defined, in the NF permeate the level of this anion is well above (more than seven-fold) the acceptable limits. In contrast, both of the NF and RO permeates clearly meet the recommended sulfate limits. The limits for phosphates and bicarbonate are not defined since the concentrations commonly found in water are not of heath or aesthetic concerns. 

Among the cations with limits generally recommended are sodium, ammonium, manganese, and iron. The levels of manganese and iron are below the detection limit, and therefore below the limits suggested. In turn, whereas sodium and ammonium levels in NF permeate (725 and 121 mg/L, respectively) are well above the limits recommended (200 and 0.5–1.5 mg/L, respectively), the levels of these cations in the RO permeate are low (18.7 and 0.1 mg/L, respectively) and compatible with drinking water standards. Vestigial elements (cadmium, lead, and mercury) are all below the detection limits in the NF and RO permeates.

In summary, the levels of several inorganic chemicals (chloride, sodium, and ammonium), pH, total dissolved solids, and conductivity in the NF permeate are not according to the recommendations and regulations for drinking-water quality. In turn, the RO permeate from dairy wastewater meets the criteria of drinking water standards, except for pH, and therefore can be recycled for cleaning processes in the dairy.

#### 3.1.2. Composition of the NF and RO Retentates 

The pH of the processed NF and RO retentates (5.34 and 5.73, respectively) falls within the appropriate range for yeast metabolism (5.0 to 6.0), as defined in standard media for yeast cultivation (Table 3, Table S2 in Supplementary Materials).

These RO and NF retentates contained, as main organic components, lactose (129–145 g/L), citric acid (15–18 g/L), proteins (14–18 g/L) and minor concentrations (<2 g/L) of galactose and lactic acid (Table 3). The lactose concentration in the retentates (129–145 g/L) is according to the requirements defined to obtain ethanol concentrations through biological conversion higher than 50 g/L, if ethanol yield is higher than 0.38 g/g (i.e., above 75% of the theoretical maximum).

When compared to the reference defined media for yeast cultivation, namely yeast nitrogen base (YNB, Difco™) or other mineral media [21,22] (Table S2), both of the NF and RO retentates contained the most relevant inorganic ions, although at different concentrations (Table 3). Generally, the level of inorganic ions in media is adjusted to the concentration of the carbon source. For example, YNB is designed for 5 g/L of carbon source, whereas “Verduyn” and “Delft” media are for 10 and 22 g/L, respectively. Accordingly, the total amount of inorganic ions in these media range from 7 to 23 g/L. The NF and RO retentates have a total of 13 to 16 g/L of inorganic ions, which is in the range of those found in standard yeast media. Because the fermentation process is to be performed at high cell density, and thus with minimal cell growth, the salts present in the NF and RO retentates should be enough to support fermentation, as long as all the necessary elements for yeast metabolism are present.

While both retentates had a surplus of chloride, sodium, potassium, and calcium, the concentration of ammonium and sulfate were approximately 10 times lower than the standard yeast media. Bicarbonate and fluoride present in retentates (respectively approximately 3300 mg/L and 130 to 373 mg/L) are not common components of these media.

The main differences in ionic composition between the NF and RO retentates were higher levels of fluoride and sodium in the NF retentate, and higher levels of chloride and potassium in the RO retentate. Sodium and fluoride are known toxic compounds for most yeast species and sodium fluoride is a common inhibitory compound for yeast metabolism at 50 mM (2 g/L) [23,24]. Moreover, potassium has been described as preferential to sodium for yeast metabolism [25]. In fact, it was reported that lactose hydrolysis by *K. marxianus* was faster in the presence of K^+^ than Na^+^ [26]. Accordingly, the RO retentate should be more advantageous as a fermentative substrate than the NF retentate because it had a higher potassium/sodium ratio (Table 3).

### 3.2. Screening of Kluyveromyces Strains for NF or RO Retentate Fermentation 

To study the ability of *Kluyveromyces* strains to directly use NF and RO retentates as a substrate for ethanol production (without supplementation), the following ten lactose-fermenting strains were selected from a yeast culture collection (PYCC): five *K. marxianus* strains and five *K. lactis* strains (Table 1). The *K. marxianus* CBS 397 (PYCC 3884) strain was expressly included in this screening for benchmarking, as this strain has been often described as one of the best lactose-fermenting yeasts, both in terms of ethanol production rate and maximum ethanol titer [27,28,29,30]. 

All strains were pre-grown on a YPD medium to generate yeast biomass, which was used to inoculate the NF and RO retentates at high cell density (4.2 ± 0.6 gCDW/L). The fermentation was performed under mild oxygen-limiting conditions (see Material and Methods for details). Fermentation processes at high cell density are faster, also favoring production of ethanol over yeast biomass and other by-products [31,32]. This is a common industrial setup in sugarcane ethanol production in Brazil, where high cell density fermentation is achieved through yeast recycling [33]. Moreover, it is generally accepted that yeast cell viability is lower under anaerobic conditions [34], and, in particular, *K. lactis* is unable to grow under strict anaerobiosis, although it can ferment and grow under oxygen-limiting conditions, e.g., below 1% air saturation [35].

**Figure 1:**
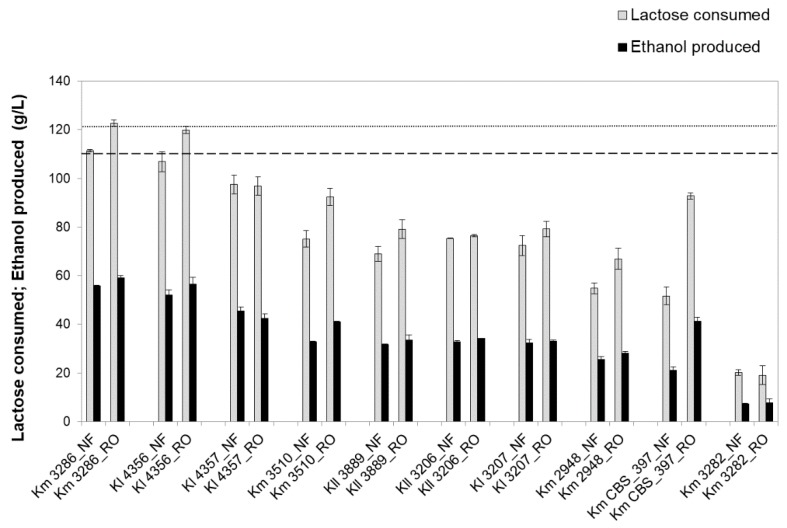
Lactose consumed (gray bars) and ethanol produced (black bars) at 16 h fermentation in the NF and RO media. Km, *K. marxianus* PYCC strain; Kl, *K. lactis* PYCC strain; Kll, *K. lactis var. lactis* PYCC strain; dash line, average initial lactose concentration in the assays in NF retentate; and dot line, average initial lactose concentration in the assays in RO retentate. Error bars represent standard deviation from the average value of three independent experiments.

Under the conditions applied, the fermentation of the NF and RO retentates with all the *Kluyveromyces* strains resulted in ethanol as the major product, with lactose/ethanol conversion yields between 67% and 93% of the theoretical maximum (Table 4). The lower ethanol yield was apparently linked to higher acetate yield. In turn, cell biomass did not significantly change during the process for all the strains. The fermentation performance of each strain in the NF or RO retentates was similar, except for the *K. marxianus* PYCC 3510 and *K. marxianus* PYCC 3884 (CBS 397) strains, which fermented more efficiently in the RO retentate than in the NF retentate (Figure 1 and Table 4).

The *K. marxianus* PYCC 3286 and *K. lactis* PYCC 4356 strains stood out as the best fermentative strains, showing lactose and ethanol conversion yields higher than 87% of the theoretical maximum, with total lactose conversion within 16 h (Table 4), performing significantly better than the others, including the benchmarking strain *K. marxianus* CBS 397. In both retentates, the *K. marxianus* PYCC 3282 strain displayed the poorest lactose fermentation capacity, with only 20% of the lactose consumed after 46 h and with the lowest ethanol yield (67% of the theoretical maximum) (Table 4). 

Lactic acid was exhaust from the NF and RO retentates in less than 46 h. As far as citric acid was concerned, the *K. lactis* PYCC 3889, PYCC 3206, and PYCC 3207 strains, as well as the *K. marxianus* PYCC 3282 strain consumed 55% to 75% of the initial amount. Interestingly, the *K. lactis* PYCC 4357, *K. marxianus* PYCC 3286, *K. marxianus* PYCC 3510, and *K. marxianus* PYCC 3884 strains produced 3 to 5 g/L citric acid. Acetic acid, glycerol (Table 4), and minor amounts (<1 g/L) of malic acid, succinic acid, and sorbitol were other end products, in addition to ethanol. Generally, the *K. marxianus* strains produced higher amounts of acetic acid (2 to 8 g/L) than the *K. lactis* strains (<1 g/L). The only exception was the *K. lactis* PYCC 4357 strain, for which the produced level of acetic acid fell in the range typically observed for the *K. marxianus* strains, i.e., approximately 2 g/L at the time of maximum ethanol (Table 4) and 3.5 g/L at 46 h. The *K. marxianus* PYCC 3884 (CBS 397) strain produced the highest amounts of acetic acid, as high as 8.3 g/L in the NF retentate (Table 4).

Glycerol was produced, in variable amounts, by all strains. High concentrations of glycerol, 2.3 to 3.0 g/L at the time of maximum ethanol (Table 4) and approximately 4 g/L at 46 h, were produced by the *K. lactis* PYCC 3889 and the *K. lactis* PYCC 3206 strains, whereas the *K. marxianus* strains, and the *K. lactis* PYCC 4356, and *K. lactis* PYCC 3207 strains produced less than 2 g/L (Table 4), even after 46 h.

Among all the ten strains examined, herein, we were intrigued by the poor fermentation features displayed by the *K. marxianus* PYCC 3282 (CBS 608) strain. In fact, other researchers had reported that this strain displays lactose consumption profiles similar to those of the *K. marxianus* CBS 397 strain, in rich medium with 20 g/L lactose, both strains being considered as good lactose consumers [36,37]. In our screening, which was performed in lactose-rich substrates (>100 g/L), the two strains showed dissimilar fermentative behaviors. The *K. marxianus* PYCC 3282 strain had the poorest performance, consuming only 20% of initial lactose (approximatley 33 g/L), whereas the *K. marxianus* CBS 397 strain was able to exhaust lactose and achieve ethanol titers up to 48 g/L in the RO retentate (Table 4). The discrepancy between our results and the literature could result from diverse tolerance to ethanol or high sugar concentration (osmotolerance). In a similar rich medium, we found that the lactose consumption rate by the *K. marxianus* PYCC 3282 strain was severely impaired when the lactose concentration was increased. At approximately 20 g/L lactose, the sugar was virtually exhausted (93% consumption) in the first 5 h of fermentation (initial 2.7 gCDW/L), while at approximately 40 g/L lactose, no sugar consumption was observed in the same 5 h period (Figure S1 in Supplementary Materials). In addition, lactose fermentation was also negatively affected by ethanol, as only 56% lactose was consumed when the medium was supplemented with approximately 20 g/L ethanol (Figure S1 in Supplementary Materials). In contrast, the *K. marxianus* CBS 397 strain was fairly insensitive to lactose concentration and ethanol. These results point to a combined effect of low tolerance of the *K. marxianus* PYCC 3282 strain to high concentrations of both sugar and ethanol.

On the basis of their fermentation performance in the NF and RO retentates, the *K. marxianus* PYCC 3286 and *K. lactis* PYCC 4356 strains were selected for further studies, still using the *K. marxianus* CBS 397 strain for benchmarking.

### 3.3. Influence of Different Oxygen-Limiting Conditions in NF or RO Rententate Fermentation Performance

The implementation of industrial fermentations of retentates from dairy wastewater processing through membrane technology requires the evaluation of oxygen availability on the fermentation performance. Taking into account that industrial fermentation processes are preferentially performed under anaerobic or severe oxygen-limiting conditions to avoid extra costs on aeration, and knowing that the *Kluyveromyces* spp. metabolism is significantly reduced under strict anaerobiosis, two different oxygen-limiting conditions (named mild and severe) were tested in the fermentation of NF and RO retentates (see Materials and Methods for details).

**Figure 2:**
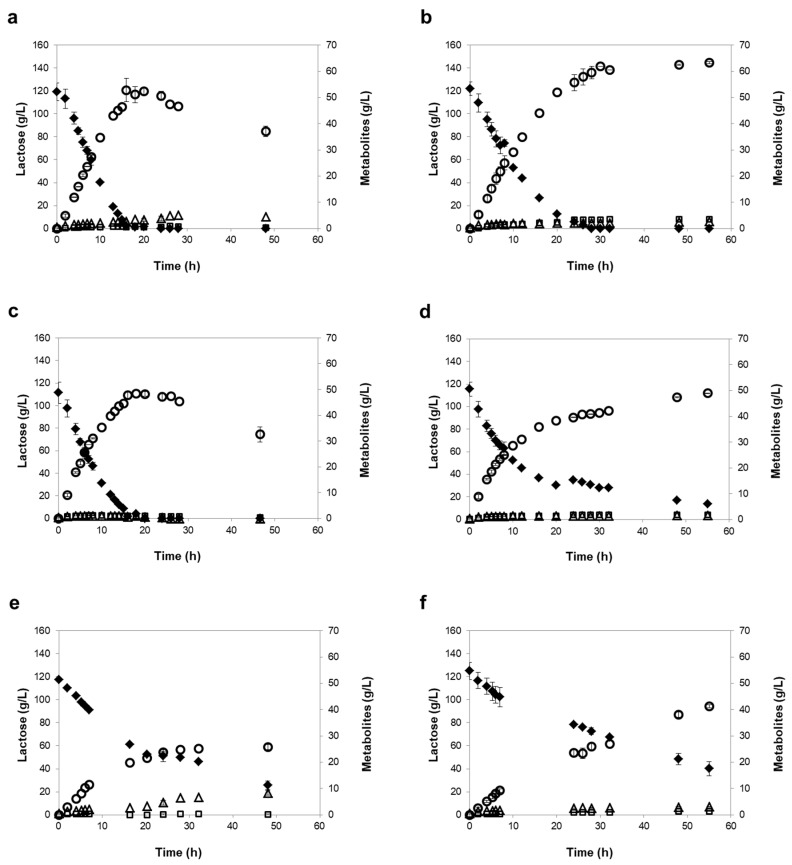
Fermentation profiles of the *K. marxianus* PYCC 3286 strain (**a**,**b**), the *K. lactis* PYCC 4356 strain (**c**,**d**), and the *K. marxianus* CBS 397 strain (**e**,**f**), in NF media, in mild oxygen-limiting conditions (left panels) and severe oxygen-limiting conditions (right panels). Lactose (♦), ethanol (◯), glycerol (□), and acetic acid (△). Error bars represent standard deviation from the average value of three independent experiments.

**Figure 3:**
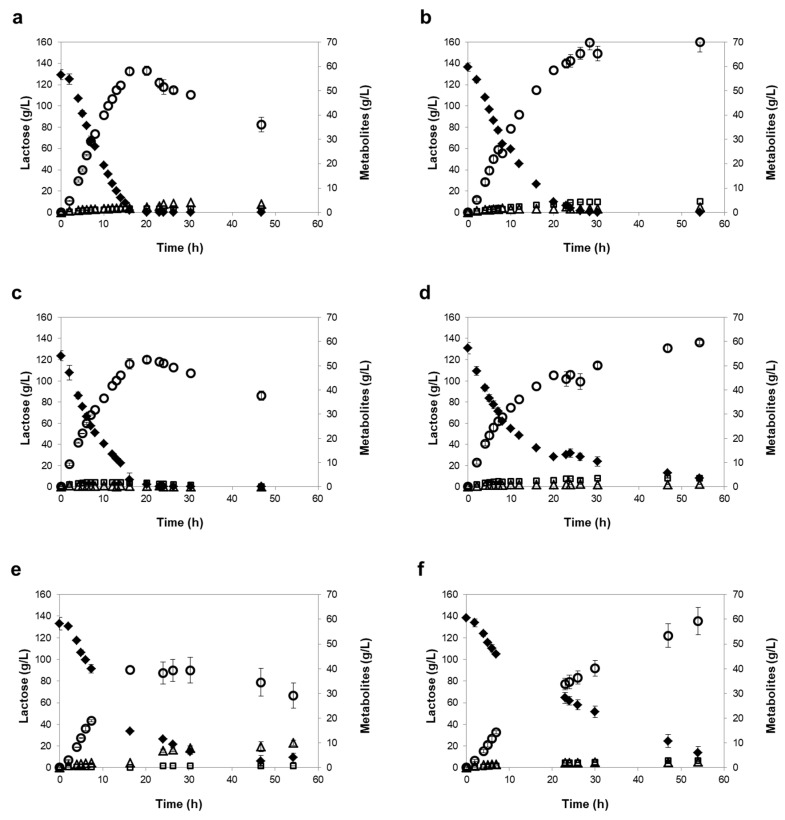
Fermentation profiles of the *K. marxianus* PYCC 3286 strain (**a**,**b**), the *K. lactis* PYCC 4356 strain (**c**,**d**) and the *K. marxianus* CBS 397 strain (**e**,**f**), in the RO media, in mild oxygen-limiting conditions (left panels) and severe oxygen-limiting conditions (right panels). Lactose (♦), ethanol (◯), glycerol (□), and acetic acid (△). Error bars represent standard deviation from the average value of three independent experiments.

The two strains selected, *K. marxianus* PYCC 3286 and *K. lactis* PYCC 4356, revealed a linear lactose consumption and ethanol production during the first 8 h of fermentation (Figure 2 and Figure 3), with higher rates under mild than under severe oxygen-limiting conditions (Table 5). Consequently, the time required to achieve maximum ethanol titers was 16 to 20 h under mild oxygen-limiting conditions and 29 to 55 h under severe oxygen-limiting conditions. The rates of lactose consumption and ethanol production were up to 25% higher in the RO retentates, which may result from the different salt composition between the two retentates. As expected, the two strains fermented slower under severe oxygen-limiting conditions, but reached higher final ethanol concentrations, because ethanol cannot be used in the absence of oxygen (Table 5). In contrast, under mild oxygen-limiting conditions, the level of ethanol decreased after lactose was exhausted (Figure 2 and Figure 3).

Under mild oxygen-limiting conditions, the *K. marxianus* PYCC 3286 and *K. lactis* PYCC 4356 strains behaved similarly. Moreover, under severe oxygen limitation, the initial lactose consumption rates were identical (Table 5), however, the *K. marxianus* PYCC 3286 strain consumed lactose at a fairly constant rate until exhaustion, while the consumption rate notably decreased for the *K. lactis* PYCC 4356 strain after the first 20 h (Figure 2 and Figure 3), this strain requiring over 55 h of fermentation to consume lactose completely.

In both of the under mild and under severe oxygen-limiting conditions, the *K. marxianus* CBS 397 strain displayed lower lactose consumption and ethanol production rates than the other two strains, especially in the NF retentates, requiring 30 to 48 h to achieve maximum ethanol titers under mild oxygen-limiting conditions and more than 55 h under severe oxygen-limiting conditions (Table 5). In the NF retentate, under mild oxygen-limiting conditions, the strain was unable to exhaust lactose (30% of lactose remains in the medium at 120 h fermentation). It is important to note that the ethanol yield and productivity values determined in our study for the reference strain *K. marxianus* CBS 397 are similar to those reported by other researchers for fermentations with this strain in synthetic medium with 200 g/L lactose and 0.2 vvm aeration (0.43 g/g and 2.12 g/L/h, respectively) [38], and in concentrated whey powder (0.44 g/g and 1.3 g/L/h, respectively) [27]. Comparable parameters were also reported for fermentation of cheese whey, despite the lower lactose content (50 to 59 g/L) [28,39]. The ethanol productivity determined for the *K. marxianus* PYCC 3286 and *K. lactis* PYCC 4356 strains under mild oxygen-limiting conditions (approximately 4 g/L/h) also compares favorably with the productivity achieved with an evolved recombinant *S. cerevisiae* strain (0.46 g/L/h), which is able to ferment concentrated cheese whey (150 g/L lactose) [40].

It was apparent that the *K. lactis* strain performed unsatisfactorily under severe oxygen-limiting conditions, confirming the known requisite of *K. lactis* strains for oxygen, probably related to impaired sterol import and lack of the Rox1p-mediated system that is essential for the response to low oxygen levels in *S. cerevisiae* [41,42]. Importantly, the performance of the *K. marxianus* PYCC 3286 strain was fairly independent of the levels of oxygen examined. This is an attractive trait of the *K. marxianus* PYCC 3286 strain, since the fermentation under severe oxygen restriction has the advantage of achieving higher final ethanol titers, as ethanol cannot be utilized via respiration, and of minimizing the need for aeration supply under industrial settings. Furthermore, this trait allows fermentations at high cell density, which may bring cost benefits to the process, especially if cell recycling can be considered. The excellent tolerance of the *K. marxianus* PYCC 3286 strain to ethanol has been recently noted by other authors who showed that this strain can tolerate up to 79 g/L of ethanol [43]. Moreover, a high beta-galactosidase specific activity was also reported for this strain (3207 IU/g DW) [44]. It is conceivable that the combination of high ethanol tolerance and high beta-galactosidase activity are decisive features for the superior fermentative behavior displayed by the *K. marxianus* PYCC 3286 strain in the NF and RO retentates, both under mild and severe oxygen-limiting conditions. Additionally, the fact that *K. marxianus* PYCC 3286 can tolerate temperatures as high as 42 °C and high salinity (16% NaCl (*w*/*v*) (CBS database (http://www.westerdijkinstitute.nl/Collections/BioloMICS.aspx) allows for a wider range of operational conditions to be tested. 

## 4. Conclusions

This work aimed to evaluate the possibility of implementing an integrated process allowing water recycling and bioenergy production from dairy wastewater. The processing of dairy wastewater through sequential crossflow UF/RO allowed the selective recovery of proteins in the UF step, while contributing to reduced fouling in the subsequent RO step. In turn, the RO processing led to a permeate that complied with legislation for water recycling and a RO retentate able to be efficiently converted into bioethanol, with lactose concentrations of approx. 150 g/L. The *K. marxianus* PYCC 3286 strain was selected as the best performing strain for the RO retentate fermentation under severe oxygen-limiting conditions, reaching 70 g/L (8.9% *v*/*v*) of ethanol, with a corresponding yield of 0.49 g/g (>95% of the maximum theoretical) and a volumetric productivity of 3.67 g/L/h.

The implementation of this integrated process under industrial settings demands further process intensification studies using this yeast strain (e.g., cell recycling), particularly in combination with long membrane technology operations to assess fouling constraints and cleaning requirements. The processing of different dairy wastewaters (e.g., those containing low lactose concentrations) may also represent new challenges for membrane operations and for yeast performance, however, this work represents an important step towards the implementation of the circular economy concept in the dairy industry.

## Figures and Tables

**Table 1:** List of *Kluyveromyces* strains used in this study.

Kluyveromyces Strains	Numbering in Other Strain Collections
K. lactis PYCC 3207	CBS 844
K. lactis PYCC 4356	
K. lactis PYCC 4357	
K. lactis var. lactis PYCC 3206	CBS 845
K. lactis var. lactis PYCC 3889	CBS 4574; DBVPG 6030; NRRL Y-2236; UCD 70-2
K. marxianus PYCC 2948	CBS 2762; ATCC 16045; CCRC 21623; DBVPG 6071; NCYC 970; NRRL YB-4327; UCD 71-13; ISA 1034
K. marxianus PYCC 3282	CBS 608; NCYC 143; NCTC 1302
K. marxianus PYCC 3286	CBS 607; ATCC 4135; CECT 1018; DBVPG 6161; JCM 3760; NCYC 6
K. marxianus PYCC 3510	NRRL Y-1122
K. marxianus PYCC 3884	CBS 397; ATCC 46537; ATCC 56497; ATCC 56752; CCRC 21477; CCRC 21698; CCT 4086; CDBB 910; CDBB 946; CECT 10584; DBVPG 6164; DSM 5422; IAM 12491; IFO 1735; JCM 22013; KCM 0230; KCTC 7152; KCTC 7155; MCYC 2712; MUCL 30017; NBIMCC 3585; NBRC 1735; NCYC 851; NCYC 1425; NRRL Y-2415; UCD 71-58; VTT C-81107; VTT C-81108

**Table 2:** Comparison of the nanofiltration (NF) and reverse osmosis (RO) permeates’ properties and composition against current recommendations and regulations for drinking water.

Parameter	NF Permeate	RO Permeate	Mandatory or Recommended Values (Table S1 ^2^)
pH (18 °C)	5.85	5.39	6.5–8.5
Total dissolved solids (g/L) (180 °C)	3.86	0.19	<0.5–0.6
Conductivity (µS/cm) (20 °C)	6440	317	2500
Anions (mg/L)			
F^-^	<0.17	<0.17	<0.5–4.0
Cl^-^	1890	78.5	<200–250 ^3^
HCO_3_^-^	105	20.7	
SO_4_^2-^	<4.0	<4.0	<250–500 ^3^
H_2_PO_4_^-^	92.4	4.8	
NO_3_^-^	4.0	<1.0	<10–50
NO_2_^-^	0.021	<0.005	<0.5–10
Cations (mg/L)			
Na^+^	725	18.7	<200 ^3^
K^+^	911	73.2	
Mg^2+^	6.2	0.2	<300 ^3^
Ca^2+^	32.9	1.0	<300 ^3^
Fe^2+^	<0.063	<0.063	<0.2–0.3 ^3^
NH_4_^+^	121	0.13	<0.5–1.5 ^3^
Mn^2+^	<0.031	<0.031	<0.05–0.1 ^3^
Vestigial Elements (µg/L)			
Cu	n.d. ^1^	n.d. ^1^	<1000–2000
Zn	n.d. ^1^	n.d. ^1^	<1000–4000
Cd	<13	<13	<3–10
Pb	<25	<25	<10–15
Hg	<0.08	<0.08	<0.5–6

^1^ n.d., not determine; ^2^ Table S1 in Supplementary Materials; ^3^ recommended values according to aesthetic acceptability thresholds.

**Table 3:** Comparison of the processed NF and RO retentates’ properties and composition against reference defined media for yeast cultivation.

Parameter	NF Retentate	RO Retentate	Reference Media (Table S2 ^2^)
Main components (g/L)			
Main Sugar	129 ± 7 (lactose)	145 ± 6 (lactose)	5–22 (d-glucose)
Galactose	1.7 ± 0.1	1.7 ± 0.2	
Citric Acid	15 ± 1	18 ± 2	
Lactic Acid	1.6 ± 0.3	0.9 ± 0.3	
Proteins	14 ± 2	18 ± 1	
pH (25 °C)	5.34 ± 0.01	5.73 ± 0.03	5.0–6.0
Anions (mg/L)			
F^-^	373	130	
Cl^-^	2254	3213	0.4–125
HCO_3_^-^	3356	3280	
SO_4_^2-^	399	382	3827–5657
H_2_PO_4_-	1938	2084	708–10262
NO_3_^-^	<5	<5	
NO_2_^-^	<0.005	<0.005	
MoO_4_^2-^	n.d. ^1^	n.d. ^1^	0.2–0.6
BO_3_^-^	n.d. ^1^	n.d. ^1^	0.5–1.9
Cations (mg/L)			
Li^+^	<0.03	<0.03	
Na^+^	2067	1139	0.1–39
K^+^	1975	4476	285–4137
Mg^2+^	178	189	493
Ca^2+^	436	442	1.2–36
Fe (total)	<0.10	<0.10	0.1–1.2
NH_4_^+^	148	222	1361–2045
Mn^2+^	<0.05	<0.05	0.2–0.5
Vestigial Elements (µg/L)			
Cu	68	31	16–127
Zn	265	203	131–2041
Co	<60	<60	0–118

^1^ n.d., not determined and ^2^ Table S2 in Supplementary Materials.

**Table 4:** Fermentation parameters in the NF and RO retentates under mild oxygen-limiting conditions. Average values of three independent experiments with corresponding standard deviation.

Strains	Time of Maximum Ethanol	Residual Lactose (g/L) ^1^	Ethanol Titer (g/L) ^1^	Acetic Acid (g/L) ^2^	Glycerol (g/L) ^2^	Ethanol Yield (g/g) ^1^
Nanofiltration (NF)						
*K. marxianus* PYCC 3286	16 h	0	56 ± 0	2.7 ± 0.2	0.7 ± 0.3	0.50 ± 0.00
*K. lactis* PYCC 4356	16 h	0	52 ± 2	0.6 ± 0.4	0.8 ± 0.1	0.49 ± 0.01
*K. lactis* PYCC 4357	20 h	4 ± 4 (6 ± 2)	49 ± 3 (46 ± 2)	1.4 ± 0.4	1.5 ± 0.2	0.48 ± 0.01 (0.47 ± 0.00)
*K. marxianus* PYCC 3510	24 h	16 ± 14 (29 ± 4)	38 ± 1 (31 ± 2)	4.2 ± 0.0	0.1 ± 0.1	0.42 ± 0.04 (0.39 ± 0.02)
*K. lactis* PYCC 3889	46 h	0 (42 ± 3)	39 ± 1 (32 ± 0)	0.5 ± 0.1	2.3 ± 0.6	0.35 ± 0.00 (0.46 ± 0.02)
*K. lactis var. lactis* PYCC 3206	24 h	20 ± 0 (38 ± 2)	41 ± 1 (33 ± 0)	0	2.8 ± 0.3	0.45 ± 0.02 (0.44 ± 0.00)
*K. lactis* PYCC 3207	24 h	8 ± 6 (31 ± 3)	42 ± 2 (32 ± 1)	0.4 ± 0.4	0.8 ± 0.3	0.44 ± 0.03 (0.45 ± 0.02)
*K. marxianus* PYCC 2948	24 h	27 ± 6 (49 ± 5)	34 ± 2 (26 ± 1)	1.7 ± 0.3	0.2 ± 0.2	0.45 ± 0.01 (0.47 ± 0.00)
*K. marxianus* PYCC 3884 (CBS 397)	46 h	32 ± 9 (65 ± 6)	26 ± 2 (21 ± 2)	8.3 ± 0.7	0	0.31 ± 0.03 (0.41 ± 0.04)
*K. marxianus* PYCC 3282	20 h	94 ± 8 (95 ± 6)	8 ± 1 (7 ± 0)	3.1 ± 0.2	0	0.36 ± 0.02 (0.36 ± 0.02)
Reverse Osmosis (RO)						
*K. marxianus* PYCC 3286	16 h	0	59 ± 1	1.8 ± 0.3	1.3 ± 0.4	0.48 ± 0.01
*K. lactis* PYCC 4356	16 h	0	57 ± 3	0.5 ± 0.4	1.7 ± 0.2	0.47 ± 0.02
*K. lactis* PYCC 4357	24 h	13 ± 5 (24 ± 1)	49 ± 4 (42 ± 2)	2.2 ± 0.2	2.6 ± 0.4	0.45 ± 0.04 (0.44 ± 0.00)
*K. marxianus* PYCC 3510	24 h	6 ± 6 (22 ± 4)	50 ± 0 (41 ± 0)	4.3 ± 0.3	0.8 ± 0.2	0.46 ± 0.01 (0.45 ± 0.02)
*K. lactis* PYCC 3889	46 h	0 (42 ± 1)	39 ± 2 (34 ± 2)	0.3 ± 0.3	2.7 ± 0.1	0.32 ± 0.01 (0.42 ± 0.00)
*K. lactis var. lactis* PYCC 3206	24 h	33 ± 5 (50 ± 2)	42 ± 1 (34 ± 0)	0	3.0 ± 1.0	0.45 ± 0.02 (0.45 ± 0.00)
*K. lactis* PYCC 3207	24 h	18 ± 12 (43 ± 7)	47 ± 0 (33 ± 1)	0.3 ± 0.3	1.0 ± 0.7	0.46 ± 0.03 (0.42 ± 0.02)
*K. marxianus* PYCC 2948	24 h	20 ±8 (51 ± 9)	44 ± 3 (28 ± 1)	1.5 ± 0.2	0.8 ± 0.0	0.45 ± 0.01 (0.42 ± 0.02)
*K. marxianus* PYCC 3884 (CBS 397)	24 h	11 ± 10 (32 ± 5)	48 ± 3 (41 ± 2)	4.3 ± 0.5	0.5 ± 0.2	0.42 ± 0.02 (0.44 ± 0.01)
*K. marxianus* PYCC 3282	20 h	111 ± 6 (112 ± 4)	8 ± 2 (8 ± 2)	2.2 ± 0.3	0.1 ± 0.1	0.39 ± 0.02 (0.40 ± 0.01)

**^1^** values at time of maximum ethanol (values in parenthesis correspond to time 16 h) and **^2^** values at time of maximum ethanol.

**Table 5:** Fermentation parameters under mild oxygen-limiting conditions versus severe oxygen-limiting conditions in the NF and RO retentates. Average values of three independent experiments with corresponding standard deviation.

Strains	Oxygen- Limiting Condition	Time of Maximum Ethanol ^1^	Consumed Lactose (%) ^1^	Ethanol Titer (g/L) ^1^	Maximum Ethanol Yield (g/g)	Ethanol Productivity (g/L/h) ^2^	Lactose Consumption Rate (g/L/h) ^2^
Nanofiltration (NF)							
*K. marxianus* PYCC 3286	Mild	16 h	98 ± 2	52 ± 4	0.47 ± 0.04	3.51 ± 0.04	8.24 ± 0.53
	Severe	30 h	100 ± 0	61 ± 1	0.49 ± 0.02	3.26 ± 0.28	7.01 ± 0.78
*K. lactis* PYCC 4356	Mild	16 h	98 ± 2	48 ± 1	0.46 ± 0.02	3.93 ± 0.05	9.01 ± 0.60
	Severe	55 h (120 h)	88 ± 1 (99 ± 1)	49 ± 0 (58 ± 1)	0.47 ± 0.02	3.19 ± 0.14	6.87 ± 0.30
*K. marxianus* PYCC 3884 (CBS 397)	Mild	48 h	78 ± 3	26 ± 1	0.28 ± 0.02	1.52 ± 0.14	3.73 ± 0.09
	Severe	55 h (120 h)	72 ± 2 (100 ± 0)	41 ± 1 (62 ± 0)	0.46 ± 0.02	1.32 ± 0.12	3.19 ± 0.20
Reverse Osmosis (RO)							
*K. marxianus* PYCC 3286	Mild	16 h	99 ± 1	58 ± 1	0.46 ± 0.02	4.18 ± 0.01	9.57 ± 0.63
	Severe	29 h	100 ± 0	70 ± 2	0.49 ± 0.01	3.67 ± 0.31	8.78 ± 0.41
*K. lactis* PYCC 4356	Mild	20 h	98 ± 2	52 ± 1	0.44 ± 0.01	4.08 ± 0.04	9.67 ± 0.60
	Severe	54 h (120 h)	94 ± 1 (100 ± 0)	60 ± 1 (67 ± 2)	0.47 ± 0.01	3.75 ± 0.25	8.30 ± 0.46
*K. marxianus* PYCC 3884 (CBS 397)	Mild	30 h	89 ± 2	39 ± 5	0.32 ± 0.04	2.74 ± 0.11	6.69 ± 0.54
	Severe	54 h (120 h)	90 ± 4 (100 ± 0)	59 ± 6 (70 ± 2)	0.48 ± 0.03	2.03 ± 0.13	4.90 ± 0.08

**^1^** values at time of maximum ethanol (values in parenthesis correspond to time 120 h), **^2^** values calculated at the first 8 h.

## Supplementary Materials

Supplementary materials can be found at https://www.mdpi.com/2076-2607/7/11/545/s1.
