# Supplementary material for: Integrated Process for Bioenergy Production and Water Recycling in the Dairy Industry: Selection of Kluyveromyces Strains for Direct Conversion of Concentrated Lactose-Rich Streams into Bioethanol
Source: Microorganisms. 2019 Nov 9;7(11):545. doi: 10.3390/microorganisms7110545 (PMC6920800; doi:10.3390/microorganisms7110545)
Supplement: Supplementary file 1 [file microorganisms-07-00545-s001.pdf]

microorganisms

MDPI

Supplementary materials

Integrated process for bioenergy production and water recycling in the dairy industry: selection of *Kluyveromyces* strains for direct conversion of concentrated lactose-rich streams into bioethanol

Maria José Leandro [1,2], Susana Marques [1], Belina Ribeiro [1], Helena Santos [2], César Fonseca [1,3,*]

Table S1. Current recommendations and regulations for drinking water.

Parameter	WHO [1]	EC [2]	EPA [3]	JWWA [4]
pH	6.5-8.5*	6.5-9.5	6.5-8.5*	5.8-8.6
Total dissolved solids (g/L) (180°C)	0.6*		0.5*	0.5
Conductivity (µS/cm) (20°C)		2500		
Anions (mg/L)				
F^-	0.5-1*	1.5	4.0/2.0*	0.8
Cl^-	250*	250	250*	200
HCO_3^-				
SO_4^{2-}	500*	250	250*	
$H_2PO_4^-$				
NO_3^-	50	50	10	10
NO_2^-	3	0.5	1	10
Cations (mg/L)				
Na^+	200*	200		200
K^+				
Mg^{2+}				300
Ca^{2+}				300
Fe^{2+}	0.3*	0.2	0.3*	0.3
NH_4^+	1.5*	0.5		
Mn^{2+}	0.1*	0.05	0.05*	0.05
Vestigial Elements (µg/L)				
Cu	2000	2000	1300	1000
Zn	4000*		5000*	1000
Cd	3	5	5	10
Pb	10	10	15	10
Hg	6	1	2	0.5

* recommended values according to aesthetic acceptability thresholds; [1] World Health Organization (**WHO**), (2017). Guidelines for Drinking-water Quality. Technical Report (https://apps.who.int/iris/bitstream/handle/10665/254637/9789241549950-eng.pdf); [2] European Commission (**EC**), (1998). COUNCIL DIRECTIVE 98/83/EC of 3 November 1998 on the quality of water intended for human consumption. Technical Report (https://eur-lex.europa.eu/legal-content/EN/TXT/PDF/?uri=CELEX:31998L0083&from=EN); [3] United States Environmental Protection Agency (**EPA**), (2017). Ground Water and Drinking Water - National Primary Drinking Water Regulations. Technical Report (https://www.epa.gov/ground-water-and-drinking-water/national-primary-drinking-water-regulations#Inorganic; https://www.epa.gov/dwstandardsregulations/secondary-drinking-water-standards-guidance-nuisance-chemicals); [4] Japan Water Works Association (**JWWA**), (2007). Supply of Drinking Water with Clean and Safe - Water Quality Standards of Drinking Water. Technical Report (http://www.jwwa.or.jp /english/water_en/water-e07.html).

Table S2. Properties and composition of reference defined media for yeast cultivation.

**Main components**	**YNB** [1]	**Verduyn** [2]	**Delft** [3]
D-Glucose (g/L)	5	10	22
pH (25°C)	5.4	5.0	6.0
Anions (mg/L)			
F^-	-	-	-
Cl^-	125	0.4	0.9
HCO_3^-	-	-	-
SO_4^{2-}	3827	3829	5657
$H_2PO_4^-$	708	2134	10262
NO_3^-	-	-	-
NO_2^-	-	-	-
MoO_4^{2-}	0.2	0.3	0.6
BO_3^-	0.5	0.9	1.9
Cations (mg/L)			
Li^+	-	-	-
Na^+	39	0.1	0.2
K^+	285	860	4137
Mg^{2+}	493	493	493
Ca^{2+}	36	1.2	2.5
Fe (total)	0.1	0.6	1.2
NH_4^+	1361	1361	2045
Mn^{2+}	0.2	0.3	0.5
Vestigial Elements (μg/L)			
Cu	16	64	127
Zn	131	1020	2041
Co	-	59	118

[1] Difco™ Yeast Nitrogen Base (without amino acids) (http://legacy.bd.com/europe/regulatory/Assets/IFU/Difco_BBL/233520.pdf); [2] Verduyn, C., Postma, E., Scheffers, W. A., & Van Dijken, J. P. (1992). Effect of benzoic acid on metabolic fluxes in yeasts: A continuous-culture study on the regulation of respiration and alcoholic fermentation. Yeast, 8(7), 501-517. doi: 10.1002/yea.320080703; [3] Jensen, N. B., Strucko, T., Kildegaard, K. R., David, F., Maury, J., Mortensen, U. H., Borodina, I. (2014). EasyClone: method for iterative chromosomal integration of multiple genes in *Saccharomyces cerevisiae*. FEMS Yeast Res, 14(2), 238-248. doi: 10.1111/1567-1364.12118

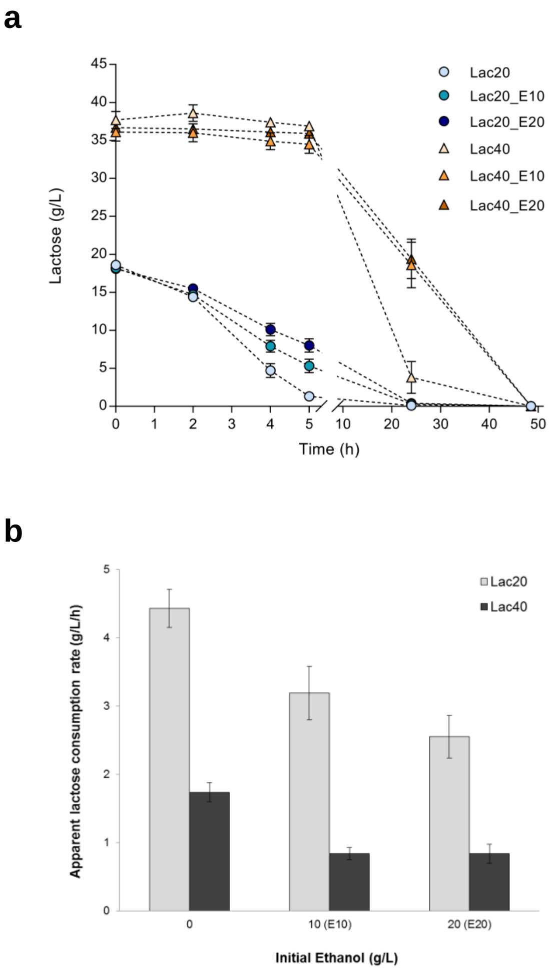

Figure S1. Effect of ethanol and lactose concentration on the lactose consumption rate by *K. marxianus* PYCC 3282. Lactose consumption by strain *K. marxianus* PYCC 3282 (CBS 608) in rich media YP with 20 g/L lactose (Lac20) and YP with 40 g/L lactose (Lac40) media, with and without addition of ethanol: initial ethanol concentrations 10 g/L (_E10) or 20 g/L (_E20). Error bars represent standard deviation from the average value of two independent experiments. **a**, Lactose consumption. **b**, apparent lactose consumption rates (Time points used for calculations: Lac20 media, from 2 h to 5 h; Lac40 media, from 5 h to 24 h). Cells were grown for 24 h in YPD medium, harvested by centrifugation (10,414 g at 4°C for 10 min), washed twice with cold sterile water and used to inoculate 10 mL of sterile medium, at an initial cell density of 2.7 ± 0.1 gCDW/L. Cells were cultivated in shake flasks (volume ratio medium/flask 1:5), with cotton plugs, in an orbital shaker (Agitorb 200, Aralab) at 30°C, with 150 rpm agitation. Assays were performed in duplicate.
